# ERα down‐regulates carbohydrate responsive element binding protein and decreases aerobic glycolysis in liver cancer cells

**DOI:** 10.1111/jcmm.16421

**Published:** 2021-03-03

**Authors:** Ying Lu, Na Tian, Lei Hu, Jian Meng, Ming Feng, Yemin Zhu, Ping Zhang, Minle Li, Qi Liu, Lingfeng Tong, Xuemei Tong, Yakui Li, Lifang Wu

**Affiliations:** ^1^ Department of Biochemistry and Molecular Cell Biology Shanghai Key Laboratory for Tumor Microenvironment and Inflammation Key Laboratory of Cell Differentiation and Apoptosis of National Ministry of Education Shanghai Jiao Tong University School of Medicine Shanghai China; ^2^ Department of Neurology Shandong Provincial Hospital Affiliated to Shandong First Medical University Shandong China; ^3^ School of Clinical Medicine Weifang Medical University Weifang China; ^4^ Cancer Institute Xuzhou Medical University Xuzhou China

**Keywords:** aerobic glycolysis, ChREBP, ERα, liver cancer, proliferation

## Abstract

Deregulated metabolism is one of the characteristics of hepatocellular carcinoma. Sex hormone receptor signalling has been involved in the marked gender dimorphism of hepatocellular carcinoma pathogenesis. Oestrogen receptor (ER) has been reported to reduce the incidence of liver cancer. However, it remains unclear how oestrogen and ER regulate metabolic alterations in liver tumour cells. Our previous work revealed that ERα interacted with carbohydrate responsive element binding protein (ChREBP), which is a transcription factor promoting aerobic glycolysis and proliferation of hepatoma cells. Here, the data showed that ERα overexpression with E2 treatment reduced aerobic glycolysis and cell proliferation of hepatoma cells. In addition to modestly down‐regulating ChREBP transcription, ERα promoted ChREBP degradation. ERα co‐immunoprecipitated with both ChREBP‐α and ChREBP‐β, the two known subtypes of ChREBP. Although E2 promoted ERα to translocate to the nucleus, it did not change subcellular localization of ChREBP. In addition to interacting with ChREBP‐β and promoting its degradation, ERα decreased ChREBP‐α–induced ChREBP‐β transcription. Taken together, we confirmed an original role of ERα in suppressing aerobic glycolysis in liver cancer cells and elucidated the mechanism by which ERα and ChREBP‐α together regulated ChREBP‐β expression.

## 1 INTRODUCTION

Hepatocellular carcinoma (HCC) usually presents substantial metabolic rearrangements.^1^, ^2^ HCC favours excessive glucose uptake and lactate production even under the condition of having oxygen, which is called aerobic glycolysis or Warburg's effect.^1^ Aerobic glycolysis supplied liver cancer cells with metabolic intermediates for anabolism to support rapid cell proliferation. The level of glucose‐6‐phosphate, which is the first product in glycolysis, is considerably increased in HCC tissues.^3^

Due to the prominent gender disparity of HCC, sex hormone receptor signalling has been involved in liver cancer pathogenesis. Oestrogen and oestrogen receptor (ER) negatively regulate initiation and progression of HCC.^4^ Among the three oestrogen receptors including ERα, ERβ and G protein–coupled ER, ERα is the dominant oestrogen receptor in hepatocytes.^5^, ^6^ There are many studies exploring the mechanism by which ERα reduces liver carcinogenesis. Oestrogen inhibits transcription of hepatitis B virus (HBV) genes and reduces rates of hepatocellular carcinoma in HBV‐infected women by up‐regulating ERα.^7^ Oestrogen reduces liver cancer risk in females by decreasing IL‐6 production by Kupffer cells.^8^ Oestrogen and ERα block metastasis of hepatocellular carcinoma cells by modulating glycogen synthase kinase 3β (GSK‐3β) and E3 ligase (β‐TrCP) expression.^9^ Moreover, it was reported that 17β‐oestradiol (E2) and ERα reprogrammed metabolism in terms of glucose usability in breast cancer cells.^10^, ^11^, ^12^ However, it remains unclear how oestrogen and ER regulate aerobic glycolysis in HCC cells.

ERα and E2 suppress lipogenesis by inhibiting mRNA and protein expression of carbohydrate responsive element binding protein (ChREBP) in insulin‐secreting INS‐1 cells.^13^ Membrane ERα signalling inhibits triglyceride synthesis through suppressing ChREBP‐α nuclear translocation.^14^ ChREBP is one of the members for the basic helix‐loop‐helix/leucine‐zipper (bHLH/ZIP) transcription factor family, mediating glucose‐regulated gene transcription.^15^ Mammalian ChREBP has two subtypes transcribed from different promoters, ChREBP‐α and ChREBP‐β. ChREBP‐α binds ChREBP‐β at the carbohydrate responsive element (ChoRE) site of its promoter and promotes its transcription.^16^ ChREBP promotes glucose utilization of liver and lipogenesis independent of insulin.^17^, ^18^ By regulating transcription of enzyme genes in gluconeogenesis, de novo lipogenesis and glycolysis, ChREBP is involved in the oetiopathogenesis of metabolic diseases and cancers.^19^ In β‐cells of islets, ChREBP induces glucose‐stimulated cell propagation.^20^, ^21^ ChREBP is crucial for the multiplication of liver cancer cell by facilitating aerobic glycolysis and anabolism.^22^

Our former work indicated that ChREBP‐α interacted with both ERα and its cofactor flightless I homolog (FLII), and FLII also interacted with ChREBP‐β.^23^ However, it remains unknown whether the ERα‐ChREBP complex regulates metabolism and proliferation of liver cancer cells. Here, we found that E2 and ERα decreased aerobic glycolysis and cell multiplication in HepG2 hepatoma carcinoma cells. ERα co‐immunoprecipitated and colocalized with both ChREBP‐α and ChREBP‐β. ERα decreased ChREBP‐α–induced ChREBP‐β transcription. Our results demonstrated ERα, ChREBP‐α and ChREBP‐β are in the same complex. The ERα‐ChREBP complex might be a potential target in the therapy of hepatoma carcinoma.

## 2 MATERIALS AND METHODS

### 2.1 Cell culture and materials

The human ChREBP‐α and ChREBP‐β cDNA was generated as described.^16^ The cDNA clones comprising different regions of ChREBP‐α and ERα were made, including ChREBP‐α 1‐251, ChREBP‐α 252‐625 and ChREBP‐α 626‐852; and ERα 1‐180, ERα 181‐282 and ERα 283‐594. Table S1 listed all the primers for above cloning. Dr Xiaoying Li at Zhongshan Hospital of Fudan University School of Medicine kindly provided the ERα plasmid. Protease inhibitor cocktail tablets (EDTA‐free) were bought from Roche (Switzerland). NP‐40 (Nonidet P‐40) and Triton X‐100 were purchased from Sigma. Dulbecco's modified Eagle's medium (DMEM) and Opti‐MEM were purchased from HyClone and Invitrogen, respectively. Foetal bovine serum (FBS) was got from Biochrom. Primary antibodies used were as follows: ChREBP (Novus; NB400‐135), HA and Myc (MBL International; M180‐3 and M047‐3), ERα (Cell Signaling Technology; 8644), GLUT2 Polyclonal Antibody (Proteintech; 20436‐1‐AP), GLUT4 Monoclonal Antibody (Proteintech; 66846‐1‐Ig), Tubulin and FLAG (Sigma; T5201 and F1804) and PARP (Invitrogen; 436400).

293T human embryonic kidney cells, human hepatocellular carcinoma cells HepG2 and SMMC7721, and HeLa human cervical cancer cells were cultured in DMEM containing 2 mmol/L L‐glutamine, 1 mmol/L sodium pyruvate, 10% FBS, 100 μg/mL streptomycin and 100 unit/mL penicillin at 37℃ in humidified 5% CO_2_ atmosphere. 17β‐oestradiol (E2) of 10 nM was used to treat cells.

### 2.2 Nuclear and cytosolic fractionation

HA‐ChREBP‐α and Flag‐ERα were transfected into 293T cells. E2 was used to treat the transfected cells. The preparation of buffer A and buffer B, and the operating steps were handled as presented previously.^23^ All the procedures were performed at 4℃.

### 2.3 Co‐immunoprecipitation

Triton X‐100 buffer contained 1% Triton X‐100, 100 mM NaCl, 40 mM Tris‐HCl, pH 8, 1 mM EDTA and 0.5% NP‐40. Using Triton X‐100 buffer to lyse fresh cells, primary antibody incubation was performed overnight at 4℃, followed by an additional incubation for 2 hours together with protein A/G agarose beads (Santa Cruz) at 4℃. Triton X‐100 buffer was applied to wash the beads for four times and then to boil the beads in 2 × SDS protein loading buffer. Then, the Western blotting analysis was carried out.

### 2.4 Luciferase assays

Flag‐ERα, HA‐ChREBP‐α, HA‐ChREBP‐β, β‐galactosidase, ChREBP‐α‐Luc reporter and ChREBP‐β‐Luc reporter were transiently transfected into 293T cells using Lipofectamine 2000 (Invitrogen). Beta‐galactosidase was employed as a reference for normalizing transfection efficiency. pcDNA3 was used to normalize the total amount of transfected DNA. At 48 hours post‐transfection, cells were collected and analysed by means of Beta‐Gal Assay Kit (Clontech) and the luciferase reporter assay system (Promega) according to the reagent specification.

### 2.5 Real‐time quantitative PCR

TRIzol (Invitrogen Life Technologies) was used to isolate total RNA from cells with or without E2 treatment in accordance with the manufacturer's recommendations. The PrimeScript™ RT Reagent Kit (Takara Bio Inc) with 10 mL assay mix containing 2 mg total RNA was applied to synthesize cDNA. The reaction mixture was kept at −20℃ until the PCR analysis. β‐actin was applied to the endogenous reference. The primer sequences of ChREBP‐α and ChREBP‐β were described previously.^16^ Table 1 listed other primer sequences.

**TABLE 1:** List of quantitative PCR primers and relevant information

Gene	Sequence (5'‐3')	Product size (bp)
ChREBP‐total		
Forward primer	AACTGGAAGTTCTGGGTGTTC	164
Reverse primer	AGGGAGTTCAGGACAGTTGG	
ERα		
Forward primer	ACCATATCCACCGAGTCCTG	194
Reverse primer	ATAGAGGGGCACCACGTTC	
β‐actin		
Forward primer	GGACTTCGAGCAAGAGATGG	234
Reverse primer	AGCACTGTGTTGGCGTACAG	

Real‐time PCR was analysed using a StepOnePlus™ Real‐Time PCR System (Applied Biosystems). Relative quantification of mRNA amount was obtained in the light of the user manual of Applied Biosystems.

### 2.6 Western blotting

Cell lysates were collected. BCA Protein Assay Kit (Pierce) was used to quantify protein concentration. Protein bands were visualized with an enhanced chemiluminescent solution (Millipore) using Amersham Imager 600 (GE).

### 2.7 Metabolic assays

Cells in 10‐cm dishes grew to about 80% confluence and then were trypsinized and resuspended in 3 mL of culture solution. An Oxytherm System (Hansatech) was applied to detect oxygen consumption.

Glucose uptake and lactate production were measured at 48 hours after cells were laid in 6‐well plates or treated, and then collected and investigated culture medium. Lactate production and glucose uptake were analysed using Lactate Assay Kit (Sigma) and Glucose Assay Kit (Shanghai Rongsheng Biotech), respectively, according to product specification.

### 2.8 Cell viability assay

A total of 2000, 4000, 6000, 10 000 or 20 000 cells were laid in 6‐well plates in triplicate. After cell adherence, CCK8 reagent was added and incubated for 2 hours, followed by OD measurement at 450 nm. The standard curve was drawn according to cell numbers and OD values. The four groups are cells stably expressing GFP or ERα cDNA with or without E2 treatment. Each group of 4000 cells were plated and cultured at 37℃ with 5% CO_2_. A number of cells were measured at days 0, 2, 4 and 6 after plating.

### 2.9 Immunofluorescent staining

Cells were grown on coverslips, rinsed twice with phosphate buffer saline (PBS), each for 5 minutes. Then, cells were treated with 4% paraformaldehyde for 15 minutes, rinsed twice with PBS, permeabilized with 0.3% Triton X‐100 for 20 minutes at room temperature and blocked in 2% goat serum for 30 minutes at room temperature and primary antibodies were incubated overnight at 4℃. Alexa 488 and 555 Fluor^®^, which were conjugated secondary antibodies, were added to the cells and reared for 30 minutes at room temperature away from light. In the final 5 minutes, DAPI (40728ES03) was added. The coverslips were mounted with nail polish after being washed twice in PBS, and images were taken using an LSM 710 laser scanning confocal microscope (Zeiss) and a confocal microscope (Leica, TCS SP8 STED).

### 2.10 Statistical analysis

Experiments were done at least three times independently; one representative experiment was displayed. Data were presented as mean ± standard deviation (SD) using Prism 5 (GraphPad Software). Student's *t* test was used to analyse the difference between the treatment group and the control group. *P* value < .05 was regarded as statistically significant.

## 3 RESULTS

### 3.1 ERα with E2 treatment reduced aerobic glycolysis and cell multiplication in HepG2 cells

**FIGURE 1:**
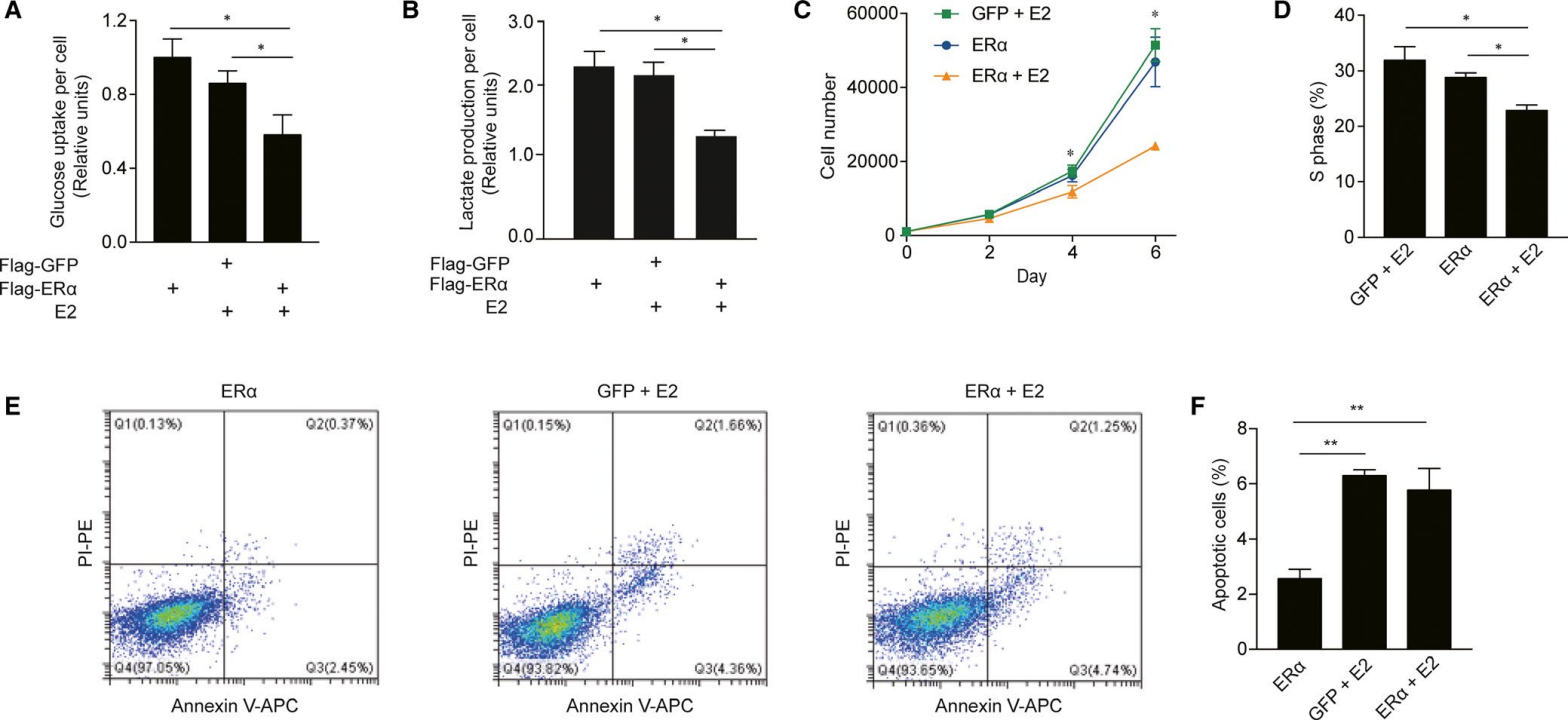
ERα overexpression with E2 treatment reduced aerobic glycolysis and cell proliferation in HepG2 cells. A, Glucose uptake, B, Lactate production of HepG2 cells stably expressing either Flag‐GFP with E2 treatment for 24 h or Flag‐ERα with or without E2 treatment for 24 h. C, Cell proliferation of HepG2 cells stably expressing either Flag‐GFP with E2 treatment for 24 h or Flag‐ERα with or without E2 treatment for 2, 4 and 6 d. D, Percentage of S phase of HepG2 cells stably expressing either Flag‐GFP with E2 treatment for 24 h or Flag‐ERα with or without E2 treatment (n = 3 biological replicates). E, Flow cytometry analysis for apoptosis of HepG2 cells stably expressing either Flag‐GFP with E2 treatment for 24 h or Flag‐ERα with or without E2 treatment. F, Percentage of apoptotic cells as indicated in E (n = 3 biological replicates). Statistical significance was calculated by unpaired Student's *t* test (mean ± SD). *P < .05, **P < .01

It was known that ChREBP enhanced aerobic glycolysis and cell multiplication in tumour cells.^22^ Previously, we found that ERα interacts with ChREBP,^23^ and we further wondered whether ERα adjusted metabolic activity and multiplication capacity of hepatoma carcinoma cells. We constructed HepG2 stable cells with Flag‐tagged GFP cDNA (Flag‐GFP) and Flag‐tagged ERα cDNA (Flag‐ERα) and compared their metabolic activity and multiplication capacity. We compared glucose uptake of Flag‐GFP with E2 treatment for 24 hours and Flag‐ERα with or without E2 treatment for 24 hours. HepG2 cells stably expressing Flag‐ERα with E2 treatment for 24 hours showed reduced glucose uptake (Figure 1A). HepG2 cells stably expressing Flag‐ERα with E2 treatment displayed lower lactate production when compared with no E2 treatment (Figure 1B). To investigate the effect of ERα overexpression with or without E2 treatment on liver cancer cell viability, cell number was counted at days 0, 2, 4 and 6 after plating. We found that the overexpression of ERα with E2 treatment slowed cell growth in comparison with the control (Figure 1C). We also examined cell cycle and apoptosis of Flag‐GFP with E2 treatment or Flag‐ERα with or without E2 treatment. ERα with E2 treatment showed a decreased percentage of S‐phase cells (Figure 1D) and increased percentage of apoptotic cells (Figure 1E,F). In brief, our findings indicate that ectopic expression of ERα with E2 treatment reduced aerobic glycolysis and cell multiplication in hepatoma carcinoma cells. SMMC7721 cells stably expressing Flag‐ERα with E2 treatment showed reduced expression of glucose transporter Glut2 and Glut4 (Figure S2B,C), which might contribute to decreased glucose uptake.

### 3.2 ERα co‐immunoprecipitated and colocalized with both ChREBP‐α and ChREBP‐β

**FIGURE 2:**
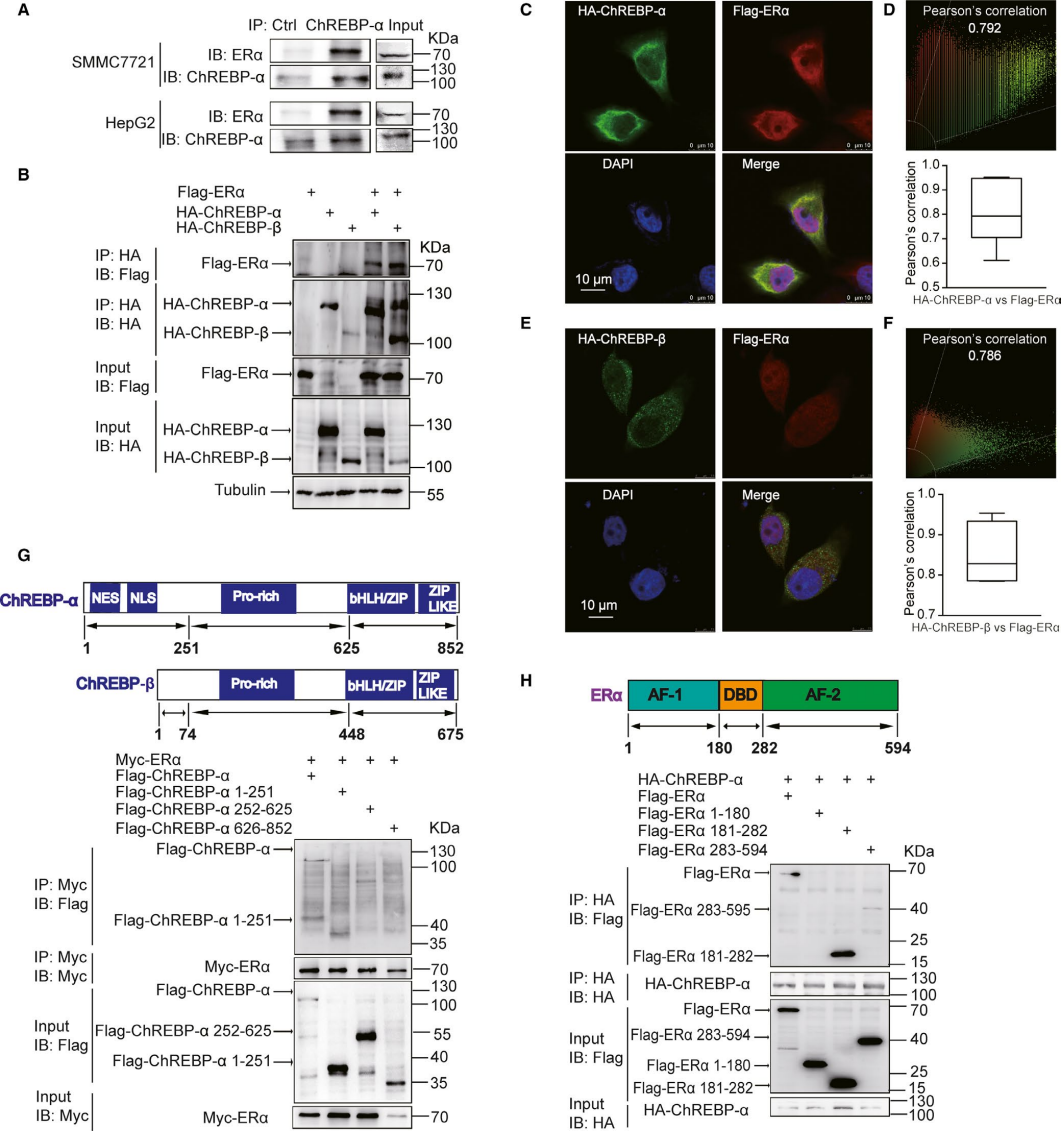
ERα colocalized and co‐immunoprecipitated with both ChREBP‐α and ChREBP‐β. A, Endogenous ERα co‐immunoprecipitated with ChREBP in HepG2 and SMMC7721 cells. B, Ectopically expressed Flag‐ERα co‐immunoprecipitated with either HA‐ChREBP‐α or HA‐ChREBP‐β in 293T cells. C, Ectopically expressed Flag‐ERα colocalized with HA‐ChREBP‐α in HeLa cells. The scale bar is 10 µm. Red: Flag‐ERα; green: HA‐ChREBP‐α; blue: DAPI. D, Upper panel: scatter plot of colocalization between HA‐ChREBP‐α and Flag‐ERα as indicated in C. Lower panel: A bar graph summarizes Pearson's correlation coefficients of HA‐ChREBP‐α and Flag‐ERα (n = 3 biological replicates). E, Ectopically expressed Flag‐ERα colocalized with HA‐ChREBP‐β in HeLa cells. The scale bar is 10 µm. Red: Flag‐ERα; green: HA‐ChREBP‐β; blue: DAPI. F, Upper panel: scatter plot of colocalization between HA‐ChREBP‐β and Flag‐ERα as indicated in E. Lower panel: a bar graph summarizes Pearson's correlation coefficients of HA‐ChREBP‐β and Flag‐ERα (n = 3 biological replicates). G, The schematic diagram shows the domain structure of ChREBP‐α and ChREBP‐β. Flag‐ChREBP‐α 1‐251, but not Flag‐ChREBP‐α 252‐625 or Flag‐ChREBP‐α 626‐852, co‐immunoprecipitated with Myc‐ERα in 293T cells. NES, nuclear export signal; NLS, nuclear localization signal; Pro‐rich, Proline‐rich domain; bHLH/ZIP, basic helix‐loop‐helix leucine‐zipper domain; ZIP‐like, leucine‐zipper‐like domain. H, The schematic diagram shows the domain structure of ERα. Flag‐ERα 181‐282 and Flag‐ERα 283‐594, but not Flag‐ERα 1‐180 co‐immunoprecipitated with HA‐ChREBP in 293T cells. AF‐1, ligand‐independent transcriptional activation function domain 1; DBD, DNA‐binding domain; AF‐2, ligand‐dependent transcriptional activation function domain 2

ERα and E2 can inhibit transcription and translation of ChREBP‐α.^13^ Our previous results showed that ectopically expressed ERα interacted with ChREBP‐α.^23^ Now, we discovered that authigenic ChREBP‐α and ERα protein also co‐immunoprecipitated in SMMC7721 hepatoma carcinoma cells (Figure 2A). Herman et al found that ChREBP had ChREBP‐α and ChREBP‐β isoforms.^16^ We also investigated whether ERα interacted with ChREBP‐β and confirmed that ERα co‐immunoprecipitated with ChREBP‐β and ChREBP‐α (Figure 2B).

In order to examine whether ERα colocalized with either ChREBP‐α or ChREBP‐β, Flag‐ERα and HA‐ChREBP‐α, Flag‐ERα and HA‐ChREBP‐β were ectopically expressed in HeLa cells and their subcellular localization was detected using immunofluorescent staining. Our data showed that HA‐ChREBP‐α or HA‐ChREBP‐β colocalized with Flag‐ERα in both cytoplasm and nucleus, respectively (Figure 2C‐F).

Next, we further analysed which domains of ChREBP‐α interact with ERα. On the basis of the structural domains of human ChREBP‐α protein, we generated three ChREBP‐α truncates including Flag‐tagged N‐terminal NES and NLS domains of ChREBP (Flag‐ChREBP‐α 1‐251), Flag‐tagged Pro‐rich domain of ChREBP (Flag‐ChREBP‐α 252‐625) and Flag‐tagged bHLH/ZIP and ZIP‐like of ChREBP (Flag‐ChREBP‐α 626‐852). We discovered that only the Flag‐ChREBP‐α 1‐251 truncate co‐immunoprecipitated with Myc‐ERα (Figure 2G). As ERα co‐immunoprecipitated with both ChREBP‐β and ChREBP‐α (Figure 2B) and ChREBP‐β did not have the N‐terminal 177 amino acids of ChREBP‐α,^16^ we deduced that ERα interacted with ChREBP‐α 178‐251, which is equivalent to ChREBP‐β 1‐74 (Figure 2H). We also constructed Flag‐tagged N‐terminal AF‐1 domain of ERα (Flag‐ERα 1‐180), Flag‐tagged DBD domain of ERα (Flag‐ERα 181‐282) and Flag‐tagged C‐terminal AF‐2 domain of ERα (Flag‐ERα 283‐595), in terms of the structural domains of human ERα protein. Our data showed that both Flag‐ERα 181‐282 and Flag‐ERα 283‐594 co‐immunoprecipitated with HA‐ChREBP‐α (Figure 2H). The discovery indicated an interaction between ChREBP‐α 178‐251 and DBD and AF‐2 domains of ERα.

### 3.3 Influence of oestrogen on the localization and expression of ERα and ChREBP‐α

**FIGURE 3:**
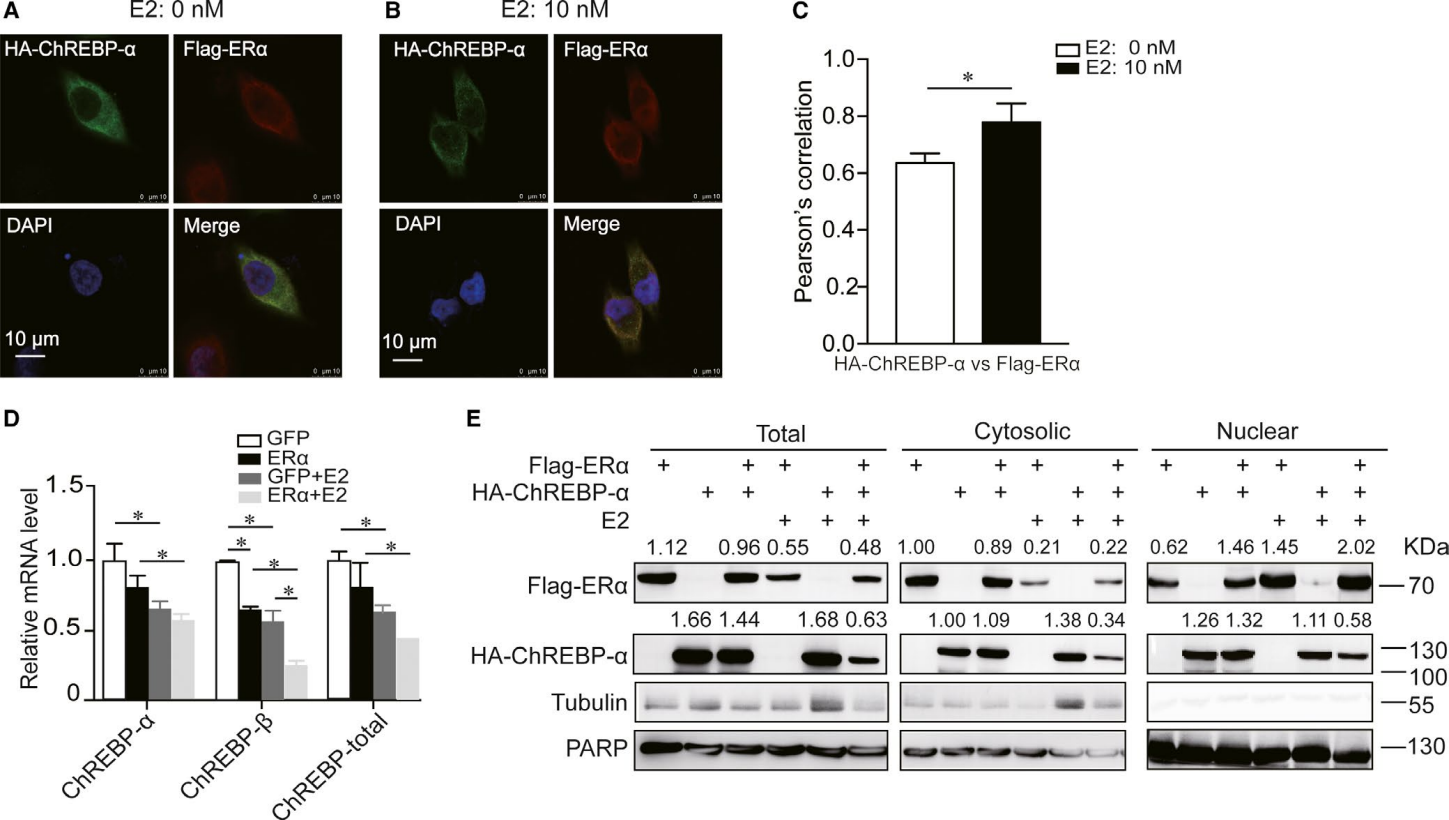
The effect of oestrogen on the localization and expression of ERα and ChREBP. A, Without E2 treatment, ectopically expressed Flag‐ERα and HA‐ChREBP‐α colocalized in both cytosol and nucleus of 293T cells. The scale bar is 10 µm. Green: HA‐ChREBP‐α; red: Flag‐ERα; blue: DAPI. B, E2 promoted the translocation of ERα from cytoplasm to nucleus without affecting subcellular localization of ChREBP in 293T cells. The scale bar is 10 µm. Green: HA‐ChREBP‐α; red: Flag‐ERα; blue: DAPI. C, Quantification of Pearson's correlation coefficients between nuclear HA‐ChREBP‐α and Flag‐ERα with or without E2 treatment. D, Real‐time PCR analysis for mRNA levels of ChREBP‐α, ChREBP‐β and ChREBP‐total at 24 h in HepG2 cells stably expressing Flag‐ERα with or without E2 treatment. E, Nuclear and cytosolic fractionation analysis showed that E2 promoted the translocation of ERα from cytoplasm to nucleus without affecting subcellular localization of ChREBP in 293T cells. Tubulin and PARP serve as loading controls for the cytosolic and nuclear fraction, respectively. Statistical significance was calculated by unpaired Student's *t* test (mean ± SD). *P < .05

We next analysed whether E2 affected the subcellular localization of ChREBP‐α and ERα. We overexpressed HA‐ChREBP‐α and Flag‐ERα in HeLa cells with or without E2 treatment and investigated the distribution of ChREBP‐α and ERα using immunofluorescent staining. Without E2 treatment, ERα was distributed in both cytoplasm and nucleus (Figure 3A). However, most of ERα translocated to the nucleus after E2 treatment and nuclear colocalization of ERα and ChREBP‐α was increased (Figure 3B,C and E). For ChREBP‐α, there was nearly no effect on its localization with or without E2 treatment (Figure 3A,B and E). There was similar effect to ChREBP‐β with E2 treatment (Figure S1).

To confirm the findings, we performed nuclear and cytoplasm separation experiment. Subcellular fractionation showed that Flag‐ERα and HA‐ChREBP‐α existed in both cytoplasm and nucleus. It is interesting that ERα increased ChREBP‐α protein level in the cytosol when there was no E2 present. However, ERα reduced ChREBP‐α protein level significantly no matter in the cytosol or nucleus when there was E2 (Figure 3E). In HepG2 cells stably expressing Flag‐tagged ERα, ERα overexpression with E2 treatment reduced mRNA levels of ChREBP‐α, ChREBP‐β and ChREBP‐total, with a more significant reduction in the ChREBP‐β mRNA level (Figure 3D). Western blot analyses showing ERα transfection and E2 treatment down‐regulated endogenous protein level of ChREBP (Figure S2A).

### 3.4 ChREBP‐α or ChREBP‐β with ERα and E2 inhibited ChREBP‐β transcription

**FIGURE 4:**
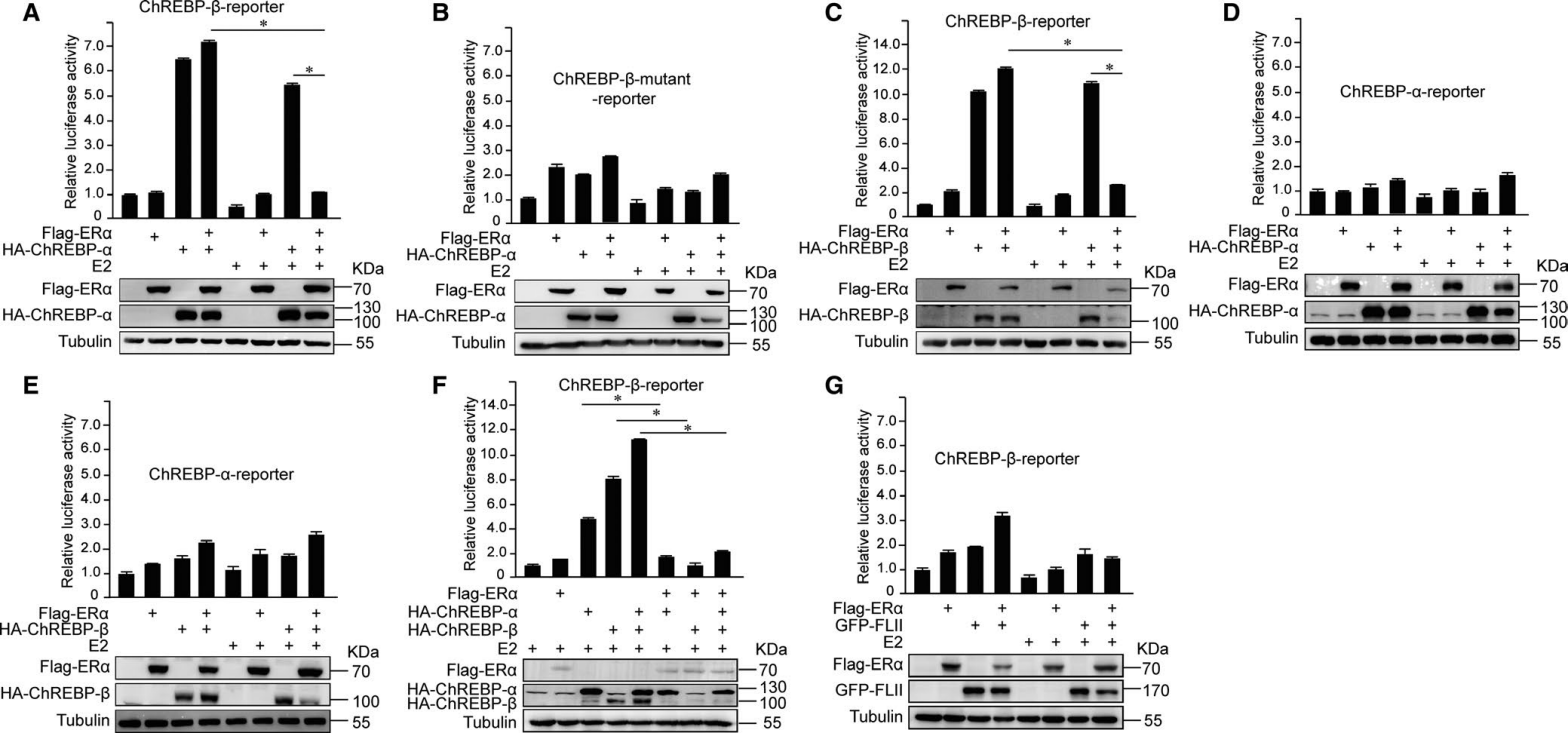
ERα and E2 reduced ChREBP‐α or ChREBP‐β–induced ChREBP‐β transcription. A, Luciferase reporter assay showed that co‐expression of ERα and ChREBP‐α with E2 treatment decreased the transcriptional activity of ChREBP‐β promoter. B, Luciferase reporter assay showed that co‐expression of ERα and ChREBP‐α with E2 treatment did not decrease the transcriptional activity of mutant ChREBP‐β promoter, which did not contain ChoRE sites. C, Luciferase reporter assay showed that co‐expression of ERα and ChREBP‐β with E2 treatment decreased the transcriptional activity of ChREBP‐β promoter. D, Luciferase reporter assay showed that co‐expression of ERα and ChREBP‐α with E2 treatment did not change the transcriptional activity of ChREBP‐α promoter. E, Luciferase reporter assay showed that co‐expression of ERα and ChREBP‐β with E2 treatment did not change the transcriptional activity of ChREBP‐α promoter. F, Luciferase reporter assay showed that co‐expression of ERα, ChREBP‐α and ChREBP‐β with E2 treatment decreased the transcriptional activity of ChREBP‐β promoter. G, Luciferase reporter assay showed that co‐expression of ERα and GFP‐FLII with E2 treatment hardly changed the transcriptional activity of ChREBP‐β promoter. Statistical significance was calculated by unpaired Student's *t* test (mean ± SD). *P < .05

Herman et al reported that one isoform of ChREBP, ChREBP‐α, transcriptionally activated the other isoform, ChREBP‐β.^16^ We next investigated whether ERα regulated the ability of ChREBP‐α to transcriptionally activate ChREBP‐β. The luciferase assay showed that ERα and ChREBP‐α or ChREBP‐β with E2 treatment sharply decreased ChREBP‐β reporter transcriptional activity (Figure 4A,C and F), but there was no significant effect on the transcriptional activity of ChREBP‐β mutant reporter (Figure 4B). Moreover, ERα and ChREBP‐α or ChREBP‐β with E2 treatment did not affect ChREBP‐α reporter transcriptional activity (Figure 4D,E). To assess whether there was specificity that ERα and ChREBP‐α or ChREBP‐β with E2 treatment reduced ChREBP‐β reporter transcriptional activity, we co‐transfected ERα and FLII to determine their effect on ChREBP‐β reporter transcriptional activity. The results revealed there was no obvious difference for the effect of ERα and FLII on ChREBP‐β reporter transcriptional activity with or without E2 treatment (Figure 4G).

**FIGURE 5:**
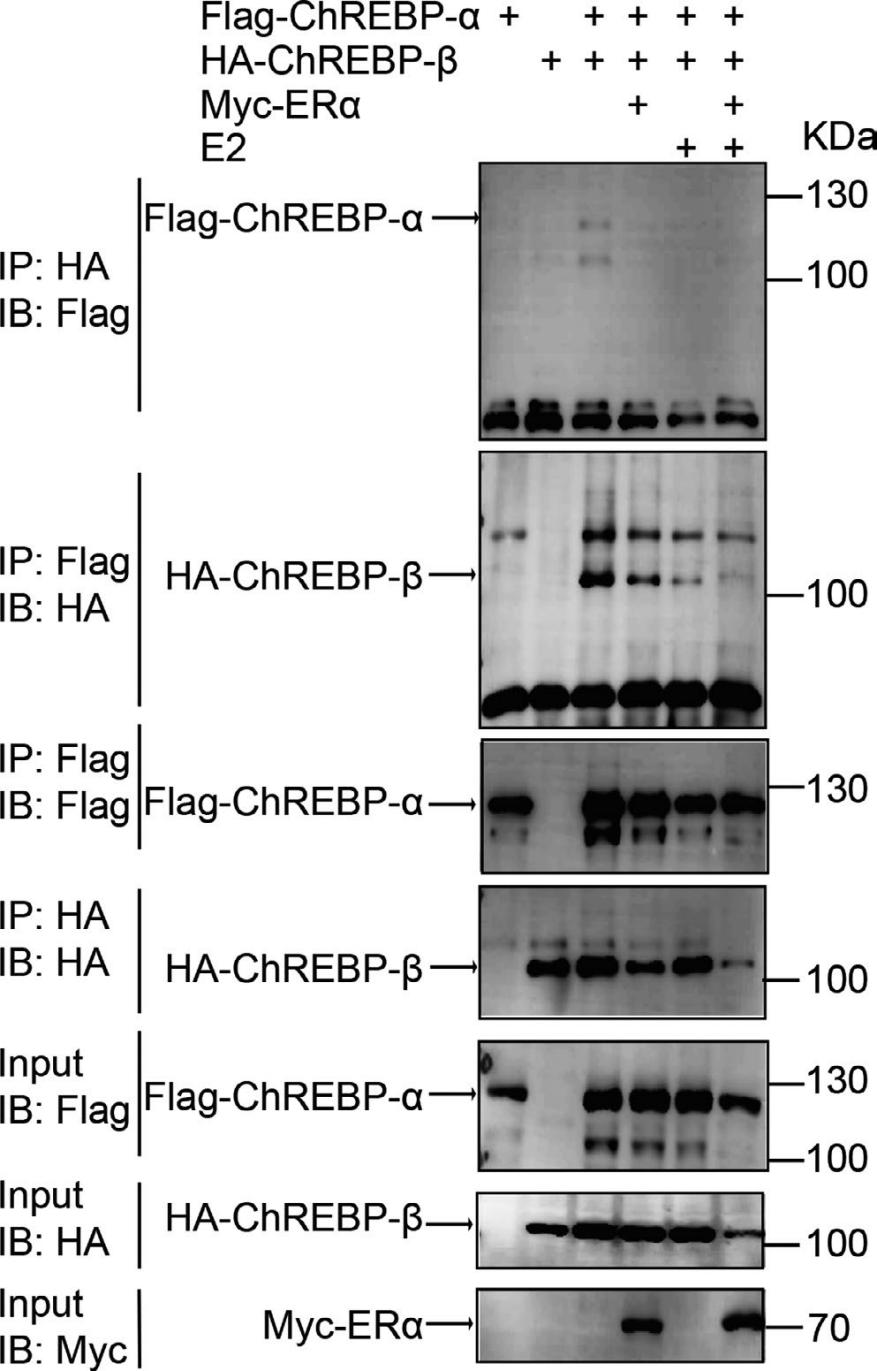
Ectopically expressed Flag‐ChREBP‐α co‐immunoprecipitated with HA‐ChREBP‐β in 293T cells. Ectopically expressed Myc‐ERα and E2 weakened the interaction. E2 treatment for 24 h

We found that ERα weakened the interaction between ChREBP‐α and ChREBP‐β, and ERα with E2 treatment further weakened the association (Figure 5). These results suggested that ERα, ChREBP‐α and ChREBP‐β might be in the same complex.

**FIGURE 6:**
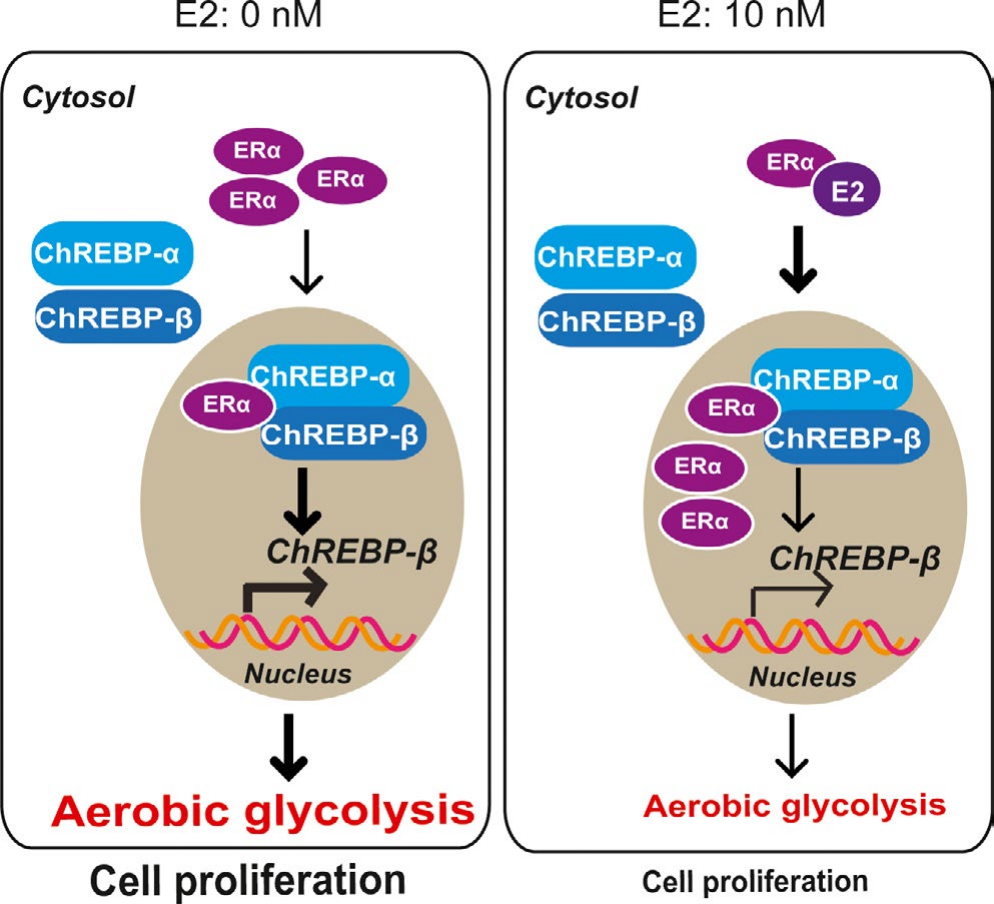
A mechanistic model showing that ERα with E2 treatment regulates aerobic glycolysis and cell proliferation in liver cancer cells by decreasing ChREBP‐β transcription

In summary, ERα with E2 treatment reduced aerobic glycolysis and inhibited cell multiplication in liver cancer cells. One of the underlying mechanisms is that ERα suppressed ChREBP activity, including ChREBP‐α–mediated ChREBP‐β transcription (Figure 6).

## 4 DISCUSSION

ERα and oestrogen negatively regulate liver cancer cell proliferation.^4^ Here, we provided a novel mechanism by showing that ERα with E2 treatment reduced aerobic glycolysis by suppressing ChREBP activity. We found ERα and oestrogen could regulate metabolism and multiplication of hepatoma carcinoma cells by reducing ChREBP protein levels. ChREBP was discovered to be a major regulator of vital genes related to glycolysis, lipogenesis and gluconeogenesis in metabolic tissues.^15^, ^17^, ^18^, ^24^, ^25^, ^26^, ^27^, ^28^, ^29^ Additionally, ChREBP facilitated the proliferation of liver and colorectal cancer cells.^22^ It was known ChREBP has two subtypes: ChREBP‐α and ChREBP‐β; ChREBP‐β is a target gene of ChREBP‐α.^16^ ChREBP reported in previous studies is actually ChREBP‐α. Our finding showed that ERα suppressed ChREBP‐α–mediated ChREBP‐β transcription provided another regulatory mechanism for the two isoforms of ChREBP.

ChREBP was down‐regulated in human breast tumour in comparison with vicinal normal tissues.^30^ Moreover, ChREBP significantly correlated with increased survival in breast cancer.^31^, ^32^ The study suggested that ChREBP might play different roles in regulating cell proliferation in breast and liver cancers. Therefore, it is worthwhile to find out why the ERα‐ChREBP axis plays distinct roles in breast and liver cancers.

ERα regulates ChREBP at both transcriptional and post‐transcriptional levels. ERα suppressed fatty acid and glycerolipid synthesis by inhibiting mRNA and protein expression of ChREBP in pancreatic islet β‐cells.^13^ Oestrogen, which opposed excessive lipid synthesis in the liver and gluconeogenesis, may partially occur from membrane ERα signalling, to suppress ChREBP and triglycerides in mature fat cells.^14^, ^33^, ^34^ Our data suggested that E2 treatment promoted nuclear translocation of ERα and dampened the binding between ChREBP‐α and ChREBP‐β. The interaction of ChREBP‐α and ChREBP‐β is indispensable for ChREBP‐α–induced ChREBP‐β transcription. Our findings mainly focused on the effect of ERα on ChREBP transcriptional activity. Going forward, a ChIP‐seq analysis of ERα should be conducted in liver cancer cell lines to investigate the direct transcriptional regulation in the promoter or enhancer of ChREBP gene.

Taken together, we uncovered a novel mechanism for ERα decreasing aerobic glycolysis and cell multiplication in liver cancer cells. The data showed that ERα, ChREBP‐α and ChREBP‐β coexisted in a complex, and ERα inhibited ChREBP‐α–mediated ChREBP‐β transcription. The ERα‐ChREBP axis might be a potential target in the therapy of liver cancer.

## CONFLICT OF INTEREST

The authors confirm that there are no conflicts of interest.

## AUTHOR CONTRIBUTIONS

**Ying Lu:** Conceptualization (equal); Funding acquisition (equal); Data curation (lead); Investigation (lead); Methodology (equal); Writing‐review & editing (equal). **Na Tian:** Data curation (equal); Formal analysis (equal); Investigation (equal). **Lei Hu:** Data curation (equal); Formal analysis (equal); Investigation (equal). **Jian Meng:** Formal analysis (equal); Investigation (equal); Methodology (equal). **Ming Feng:** Formal analysis (equal); Investigation (equal); Software (equal). **Yemin Zhu:** Data curation (equal); Formal analysis (equal); Investigation (equal). **Ping Zhang:** Data curation (equal); Formal analysis (equal). **Minle Li:** Data curation (equal); Formal analysis (equal). **Qi Liu:** Data curation (equal); Investigation (equal). **Lingfeng Tong:** Formal analysis (equal); Investigation (equal). **Xuemei Tong:** Conceptualization (lead); Funding acquisition (lead); Project administration (equal); Resources (lead); Writing‐review & editing (lead). **Yakui Li:** Data curation (equal); Funding acquisition (equal); Formal analysis (equal); Investigation (equal); Software (equal). **Lifang Wu:** Conceptualization (lead); Data curation (lead); Funding acquisition (equal); Investigation (lead); Project administration (equal); Supervision (lead); Writing‐original draft (lead); Writing‐review & editing (lead).

## Supporting information

Fig S1

Fig S2

Table S1

## Data Availability

The data that support the findings of this study are available from the corresponding author upon reasonable request.
