# Supplementary figures and images for: ERα down‐regulates carbohydrate responsive element binding protein and decreases aerobic glycolysis in liver cancer cells
Source: J Cell Mol Med. 2021 Mar 3;25(7):3427–36. doi: 10.1111/jcmm.16421 (PMC8034478; doi:10.1111/jcmm.16421)

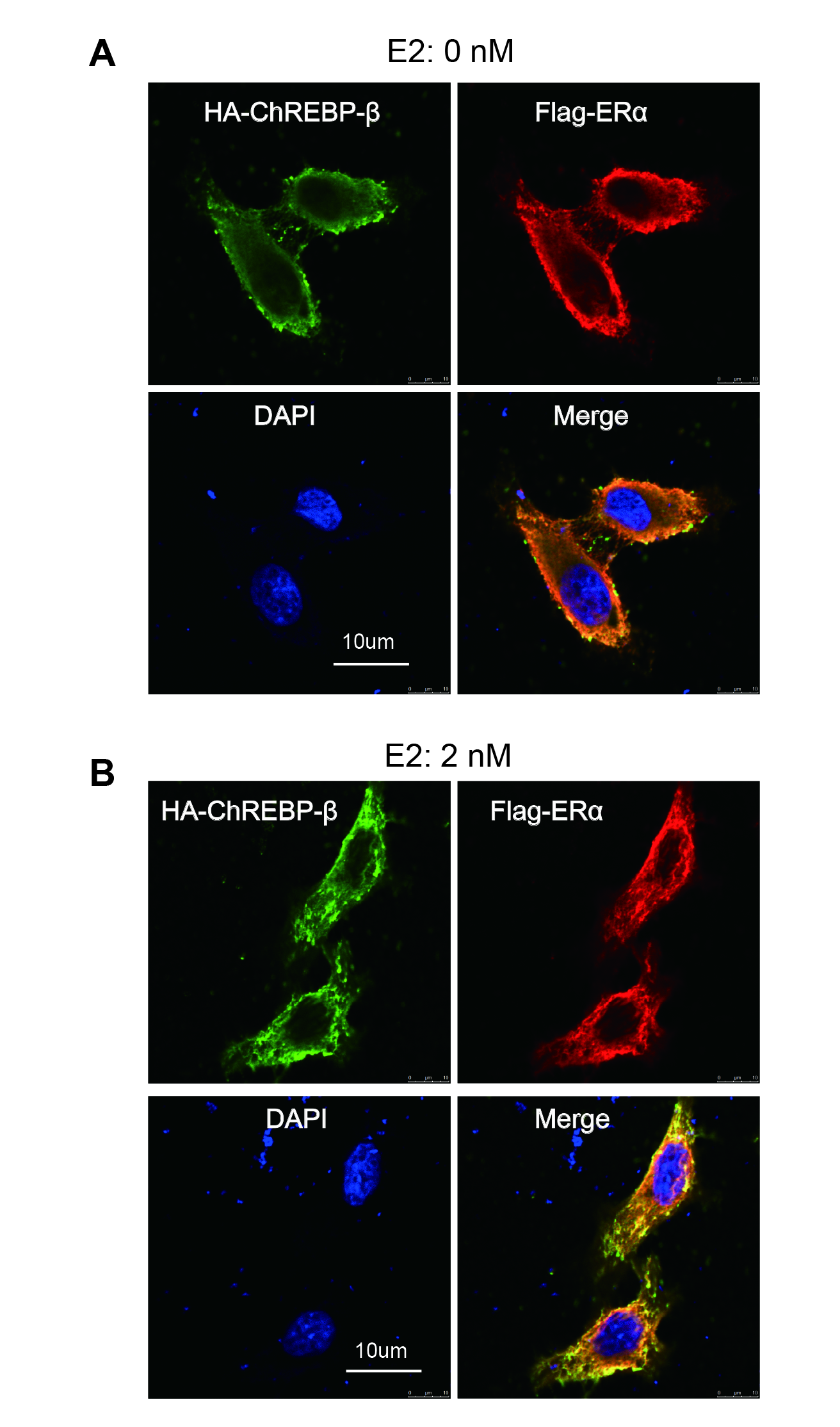

Supplement: Supplementary file 1 — Fig S1 [file JCMM-25-3427-s002.tif]

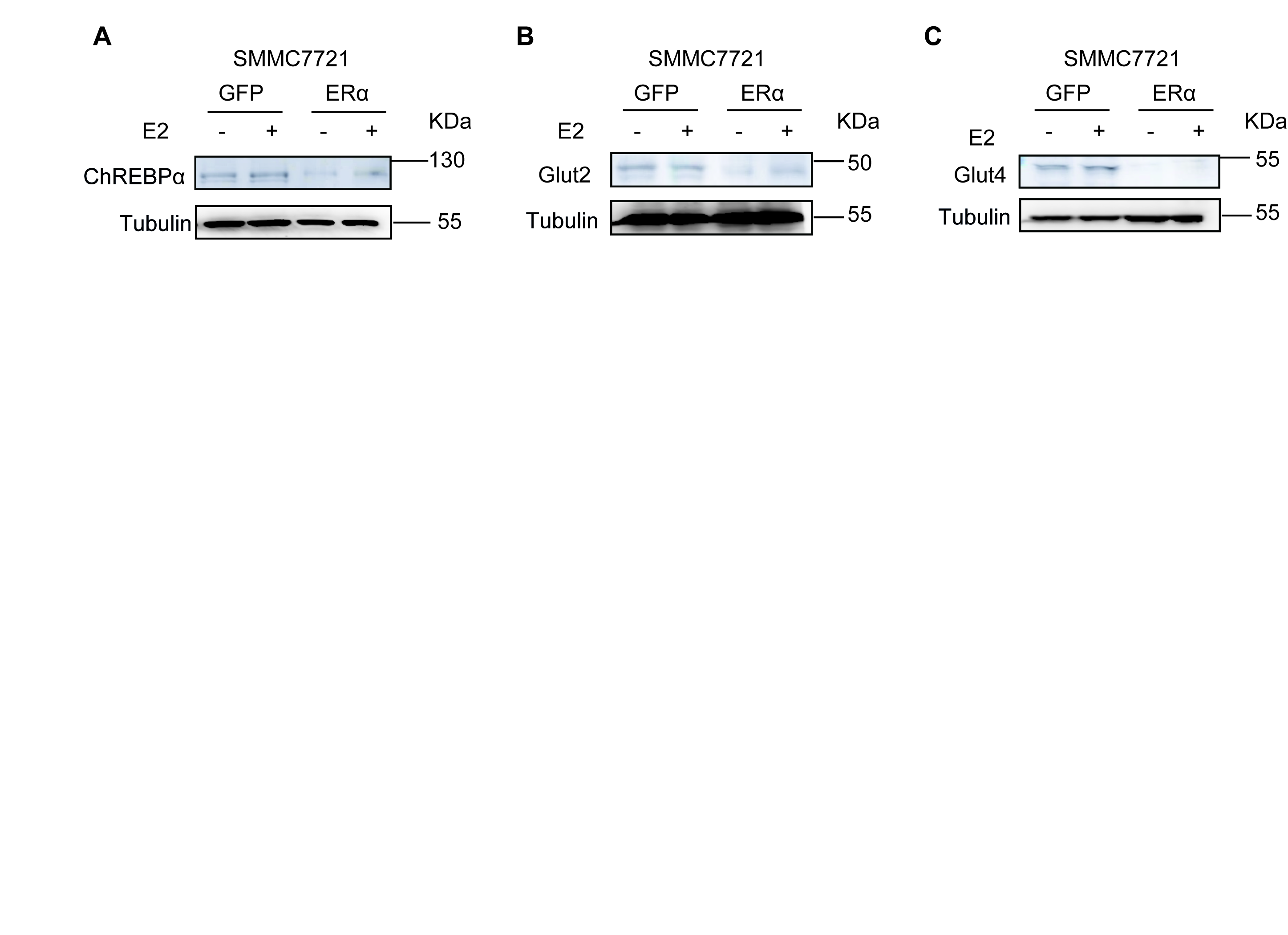

Supplement: Supplementary file 2 — Fig S2 [file JCMM-25-3427-s003.tif]
