# Supplementary material for: ERα down‐regulates carbohydrate responsive element binding protein and decreases aerobic glycolysis in liver cancer cells
Source: J Cell Mol Med. 2021 Mar 3;25(7):3427–36. doi: 10.1111/jcmm.16421 (PMC8034478; doi:10.1111/jcmm.16421)
Supplement: Supplementary file 3 — Table S1 [file JCMM-25-3427-s001.docx]

**Table S1 The primer sequences for cloning the truncations of ChREBP and ERα**

| **Gene** |  | | **Sequence(5'-3')** |
| --- | --- | --- | --- |
| ChREBP-α 1-251 | Forward | TTATAGGATCCGGCCGGCGCGCTGGCA | |
|  | Reverse | GCGCCGCTCGAGCTAGTCGGACAAAAAGC | |
| ChREBP-α 252-625 | Forward | ATCGCGGGATCCATCTCAGACACTCTCTTC | |
|  | Reverse | ATGCAGCTCGAGAATAGTCCCAGGGCCTG | |
| ChREBP-α 626-852 | Forward | ATCATGGGATCCCTGAGCGTCCGTGTCTCT | |
|  | Reverse | CGCGCGCTCGAGCTATAAAGGTTTGCCAAG | |
| ERα1-180 | Forward | ATG ACC ATG ACC CTC CAC ACC AAA G | |
|  | Reverse | CTT GGC AGA TTC CAT AGC CAT ACT | |
| ERα181-282 | Forward | GAG ACT CGC TAC TGT GCA GTG T | |
|  | Reverse | AGA CCC CAC TTC ACC CCT GC | |
| ERα 283-594 | Forward | GCT GGA GAC ATG AGA GCT GCC AA | |
|  | Reverse | TCA GAC CGT GGC AGG GAA ACC CT | |
